# Business ethics of private general practitioners in KwaDukuza, KwaZulu-Natal

**DOI:** 10.4102/phcfm.v2i1.26

**Published:** 2010-03-04

**Authors:** Indiran Govender, Gary Morris

**Affiliations:** 1 Department of Family Medicine and Primary Health Care, University of Limpopo, Medunsa campus, South Africa

**Keywords:** stress, over-servicing, general practitioners, kickbacks, medical misconduct

## Abstract

**Background:**

Private general practitioners (GPs) have been criticised by the lay press citing unethical practice and the acceptance of kickbacks. In 2003, the Ethics Institute of South Africa conducted a national study of all doctors and also accused private GPs of unethical practice. In countries such as South Africa, with a practice of fee-for-service payments, there may be a temptation to put material interests above the best interests of patients. Private GPs, on the other hand, are of the opinion that the press and the Ethics Institute publication have unfairly singled them out.

**Objective:**

To detect whether private GPs in KwaDukuza perceive their colleagues to be practising ethically.

**Method:**

The study entailed a cross-sectional descriptive study design, in which all 30 private GPs based in KwaDukuza, KwaZulu-Natal, were asked to complete a self-administered questionnaire during 2003. The survey was done on a voluntary basis and anonymity and confidentiality was maintained.

**Results:**

Twenty-five doctors returned completed questionnaires (an 83.3% response rate). Seventy per cent perceived their peers to be practicing ethically, while 48% (12/25) reported that they did not observe any medical misconduct by their colleagues. The majority of the respondents (76%) reported that they did not know of any colleague who supplemented his or her income through the over-servicing of patients. The majority of the respondents (84%) also reported that their colleagues never accepted cash payments that were not declared for income tax purposes. Medically unnecessary tests are a form of unethical behaviour pertaining to over-servicing, and 64% of the respondents reported that medically unnecessary tests to satisfy patient requests were not an important reason for performing these tests. The doctors expressed high stress levels from multiple stressors in their occupation.

**Conclusion:**

GPs in KwaDukuza indicated that they were under stress, but still practised ethically. The GPs emphasised the need for more training in medical ethics at all levels of the medical career. The majority of GPs of KwaDukuza perceive their colleagues to be practising ethically.

## INTRODUCTION

Private general practitioners (GPs) in South Africa have been criticised by the lay press of taking kickbacks and of unethical practice. The Ethics Institute of South Africa has added scientific influence to this accusation by carrying out a national study among all doctors^1^. The South African Revenue Service has used this information to target private practitioners. The findings of Landman and Mouton, who conducted the Ethics Institute's study, show that two-thirds of doctors surveyed indicated that they have observed medical misconduct by a colleague.^1^ Two-thirds of the respondents in the survey indicated that doctors supplemented their income by over-servicing their patients. Over-servicing as an ethical misconduct occurs when doctors recommend or perform medically unnecessary tests to benefit the doctor financially.

Paying a doctor for his or her professional services is sometimes viewed with resistance by society.^2^ The patient, the medical aid society and market factors all influence the remuneration of the doctor. Business-related issues also influence doctors in private practice. Doctors in private practice are faced with two competing interests: that of their ethical duties to their patients and that of the business interests of the practice.^3^

The rise of healthcare consumerism is seen to compromise the beneficence of the doctor-patient relationship.^4^ Some moralists argue that beneficence can be an effective means of cost control as well as a crucial component of the moral foundation of the medical profession.^4^ However, other South African doctors have pointed out that in today's medical practice, bottom-line profit is crucial to the survival of a private practice.^5, 6, 7^ Hillier reports that in countries such as South Africa, with a practice of fee-for-service payments, there may be a temptation to put material interests above the best interests of patients.^2^

Private GPs feel they have been unfairly criticised. The ethical behaviour of doctors is a controversial issue. The researchers were interested in determining the perceptions of private GPs in KwaDukuza (formerly known as Stanger), of the ethical behaviour of their colleagues.

At the time of the study, there were 31 GPs (including the researcher) in Kwadukuza, which is located on the north coast of KwaZulu-Natal.

## METHOD

A quantitative, cross-sectional, descriptive study, using Landman and Mouton's questionnaire,^1^ was carried out involving all the GPs in KwaDukuza. The questionnaire used was a validated and reliable tool, used in a similar study by Landman and Mouton^1^. Four questions in the questionnaire were repeated in different forms in different parts of the questionnaire to check for truthfulness of the responses. This study compared results to the survey conducted by Landman and Mouton, on behalf of the Ethics Institute of South Africa.^1^ The questionnaire used by the Ethics Institute of South Africa was developed over a period of six weeks, in consultation with various experts in the field of medical ethics and survey research.^1^

### A methodological note on ethics research

Empirical studies, and surveys in particular, on ethics are difficult. They fall within the general category of sensitive research.^1^ It is considered sensitive research for a number of reasons, but primarily because of the degree of ‘reactivity’ involved. Reactivity refers to the phenomenon in social research where research subjects and participants react and respond in various ways because they are aware that they are being investigated (except in cases of covert research), which in turn can affect the overall validity and reliability of data.^1^

Some of these responses involve lying, deception, social desirability responses (trying to please the investigator) and acquiescence response sets (agreeing with everything being asked). Reactivity is a general feature of much social research but is more pronounced in research on sensitive topics. Any study that addresses matters of morality (such as this survey), private actions and behaviours (for example sexuality), or potentially threatening issues (for example information on income or crime-related behaviours) is generally considered sensitive research. In studies of this nature the reactivity is often even more pronounced. This clearly requires greater awareness and methodological sensitivity.^1^

In formulating the items in the questionnaire that refer to matters of unethical behaviour, the first problem Landman and Mouton^1^ faced was that no respondent would intentionally incriminate themselves when required to answer affirmatively about an unethical action. This led to the formulation of questions in the third person. Rather than asking the question: ‘Has a private hospital offered you a kickback for referring patients to the hospital?’ questions were rephrased to read: ‘How often – in your estimate – does it happen to a doctor that he or she refers a patient to a private hospital and then gets a financial incentive from that hospital?’

A pilot study was also carried out by this study's researchers on five GPs from another town. This showed that all understood the questionnaire and that it took on average 12 minutes to complete. The survey was done on a voluntary basis and anonymity was maintained.

Thirty private GPs in KwaDukuza were sampled and 25 responses were received (giving a response rate of 83.3%). To encourage the GPs’ response and to prevent sample bias, one of the researchers telephoned the doctors prior to them receiving the questionnaires, to explain the purpose of the study to them and to encourage all doctors to participate. After two weeks, reminders were sent to all doctors with a copy of the questionnaire.

The courier from the local pathology laboratory was used to distribute and collect the questionnaires. The distribution and collection of questionnaires took place in October 2003.

Ethics approval for the study was obtained from the MEDUNSA research committee.

## LIMITATIONS

This survey was carried out among the GPs in KwaDukuza only, and it is possible that generalisations may not be made to other doctors or to other geographic areas of South Africa. Another limitation of the study is that it measures the reported perceptions of others’ practice and not actual practice.

It is possible that the GPs that responded to the survey may be different from the non-respondents. Those GPs responding may have felt a need to defend their colleagues and profession. The possible biases in the GPs that responded may include wishful thinking, protective biases towards their medical profession and personal image and the so-called group think, which may result in misconceptions.

## RESULTS

The results of this study may not be generalisable to doctors in other fields of practice or to GPs in other geographic areas of practice, because this study was limited to GPs in KwaDukuza.

Confidence intervals for the percentage response (95%) are shown for the results (Table 1), however, the sample size was small and therefore no clear statistical relationships could be drawn.

**TABLE 1:** Doctors’ need for ethics in continuing professional development (CPD), perceptions of medicine as a unique profession and of colleagues’ ethical conduct

Statement	Strongly agree	Agree	Neutral	Disagree	Strongly disagree
There is a need for more ethics education	7 (28%) CI 12–49%	6 (24%) CI 9–45	6 (24%) CI 9–45	5 (20%) CI 7–41	1 (4%) CI 0–20
The practice of medicine imposes a higher standard of moral integrity than other professions	11 (44%) CI 24–65	13 (52%) CI 31–72	1 (4%) CI 1–26	0 CI 0–14	0 CI 0–14
The vast majority of doctors (more than 80%) are ethical in their professional conduct	9 (36%) CI 18–57	9 (36%) CI 18–57	4 (16%) CI 5–36	3 (12%) CI 3–31	0 CI 0–14

Table 1 shows that 72% of the responding GPs perceived the majority of doctors to be practising ethically.

Six GPs knew of colleagues that supplement their income through the over-servicing of patients (Table 2).

**TABLE 2:** Unethical practices that GPs may conduct to enhance their remuneration

Question	Yes	No	Don't know
Do you know GPs that supplement theirincome through the over-servicing of patients?	6 (24%) CI 9–45	13 (52%) CI 31–72	6 (24%) CI 9–45
Do you know GPs that supplement their income through specialists?	1 (4%) CI 1–26	18 (72%) CI 51–88	6 (24%) CI 9–45
Do you know GPs that supplement their income through private hospitals/clinics?	5 (20%) CI 7–41	13 (52%) CI 31–72	7 (28%) CI 12–49

The majority of GPs (84%) stated that their colleagues never increase charges to the medcial aid/insurance by over-servicing. Two doctors thought that doing medically unnecessary tests to satisfy the patient's request was a very significant pressure.

Almost all GPs reported stress from multiple sources. The important stressors included inadequate remuneration (96% of respondents), government intervention (100%), managed care (92%) and fear of litigation (72%).

## DISCUSSION

The results show that the majority of KwaDukuza GPs perceive their colleagues to practice ethically (72%) in a very uncertain and stressful private practice environment. This is in contrast to lay press reports.^8^ Kickbacks, fee-splitting, over-servicing and fraudulent claims are contrary to the codes of conduct and regulations of the medical profession and also illegal in terms of South African law. All results obtained in this study are within the 95% confidence interval. The wide confidence intervals are due to the small sample size. Although this implies statistical significance, the small size must be taken into account when interpreting the results. The specific responses to questions will now be discussed.

### Do most doctors act ethically?

Eighteen (72%) of the GPs stated that doctors act ethically (Table 1). In Landman and Mouton's study, 73% of the doctors were of the opinion that the vast majority of South African doctors are ethical in their professional conduct, while 10% of the doctors disagreed.^1^

This indicates that most of the doctors of South Africa are of the opinion that the vast majority of doctors are ethical in their professional conduct. No other research on this issue has been done in South Africa, therefore the evidence could suggest that the majority of doctors perceive their colleagues to be practising ethically.

### Continuing medical education on ethics

The results are similar to the national study^1^ as far as the need for more ethics education and a larger emphasis of ethics in the continuing professional development (CPD) plan of the Health Professions Council of South Africa (HPCSA) are concerned. The local Independent Practitioner Association may address this need for more ethics training. The HPCSA may have to consider the results of the two surveys and increase the ethics weight of the CPD programme.

### Medicine imposes a higher standard of moral integrity on the doctor

Almost all doctors (96%, Table 1) from KwaDukuza strongly agreed that a medical career imposes a higher moral integrity than other professions. This is necessary for doctors to evoke a trusting relationship with their patients. To agree that their career imposes stronger moral integrity and commitment is a basis for sound ethical practice. The changing nature of the medical profession and the new demands being placed on medical practitioners as a result of business-type considerations require new innovative approaches. South African doctors are increasingly faced with these new developments and are clearly divided over many of the implications. In the national study^1^ there are equal proportions of doctors who believe that a medical practice is merely another business and doctors who believe that it is different from other occupations. The KwaDukuza GPs, on the other hand, clearly indicated that medicine as a profession is unique, and may be regarded as a calling. The GP in private practice has the autonomy of a self-employed professional; however, this entails both opportunity and responsibility. This responsibility includes ethical and legal behaviour by the doctor.

The majority of KwaDukuza respondents (84%, Table 3) stated that their colleagues almost never accept cash payments from patients that they do not declare for income tax purposes. This response is different from the national study,^1^ which included a large proportion of state-employed doctors who stated that their colleagues frequently accept cash payments and that these are not declared for income tax purposes. Most of the doctors in this study (84%) indicated that all cash received is declared for tax purposes and doctors do pay their correct taxes. Tax avoidance, as opposed to tax evasion, is not illegal. It does however affect the image and standing of the medical profession as a whole if members are seen to be avoiding tax and it therefore undermines the trust in the profession. This study showed that these GPs did not avoid or evade tax.

**TABLE 3:** GPs accepting cash payments and over-servicing patients to supplement their income

Question	Daily	Weekly	Monthly	Never
In your opinion, how often does a GP accept cash payments that are not declared for tax purposes?	3 (12%) CI 3–31	1 (4%) CI 1–26	0 CI 0–14	21 (84%) CI 64–95
In your opinion, how often does a GP increase charges to the medical aid insurance by over-servicing?	1 (4%) CI 1–26	1 (4%) CI 1–26	2 (8%) CI 1–26	21 (84%) CI 64–95

Over-servicing as an ethical misconduct occurs when doctors recommend or perform medically unnecessary tests to benefit the doctor financially.^9^ Some of the activities that are fraudulent include over-servicing, billing for services not rendered and the exchange of goods/services for medically coded procedures/investigations. The GPs of KwaDukuza are commendable, as they are perceived by their colleagues as not performing unnecessary tests to benefit themselves. This implies that only clinically indicated and necessary tests are done for the benefit of the patient. Only 36% of respondents from Table 4 indicated that some colleagues perform unnecessary tests to satisfy the demands of patients. Unnecessary investigations/interventions/referrals may be done for reasons that are not justified based on medical need or on evidence-based practice, but these may not necessarily be illegal. These may be done due to pressure from patients, the doctor's anxiety not to miss a diagnosis or the doctor's fear of litigation. Patients in private practice can sometimes exert considerable demands to have medical tests done. However, the majority of the GPs are able to withstand this pressure.

**TABLE 4:** Importance of unnecessary medical tests and unwarranted medical certificates

Question	Not important at all	Unimportant	Important	Very important
How important is it to do medically unnecessary tests to satisfy the patient?	9 (36%) CI 18–57	7 (28%) CI 12–49	7 (28%) CI 12–49	2 (8%) CI 1–26
How important is it to issue unwarranted medical certificates to benefit financially?	11 (44%) CI 24–65	13 (52%) CI 31–72	1 (4%) CI 1–26	0 CI 0–14
How important is it to issue unwarranted medical certificates to satisfy the patient?	13 (52%) CI 31–72	6 (24%) CI 9–45	5 (20%) CI 7–41	1 (4%) CI 1–26

### Medically unwarranted certificates

KwaDukuza GPs were asked to rate various reasons for issuing medically unwarranted certificates according to how important reasons were to them.

### Unwarranted medical certificates to satisfy patients

It has been proposed by the national study^1^ that doctors, for different reasons, sometimes issue medical certificates that are medically unwarranted. From Table 4 it is seen that 19 GPs, or 76%, indicated that they believe that issuing unwarranted medical certificates to satisfy the patient was either not important at all (52%) or unimportant (24%). Only 24% (6) of the GPs indicated that the issuing of unwarranted sick certificates to satisfy the patient was important.

The national study by Landman and Mouton^1^ showed that the majority of doctors in their sample (60%) indicated that they believe that keeping the patient satisfied is a sufficiently important consideration when issuing medical certificates, even if the certificate is not warranted. A noteworthy fact is that 40% of the Landman and Mouton sample^1^ regards this consideration as unimportant.

In comparing the two surveys, GPs in KwaDukuza determinedly indicated that issuing unwarranted sick certificates to satisfy the patient is not an important enough reason for them to be coerced into issuing medical certificates.

### Unwarranted medical certificates to benefit financially

In the sample of GPs from KwaDukuza, it was emphatically stated (96%) that the issuing of unwarranted sick certificates for financial benefit almost never occurs (refer to Table 4). Only one GP believes that this might be an important reason to issue a sick certificate. This indicates that KwaDukuza GPs are unlikely to issue sick certificates just to benefit financially.

In the national study, 81% indicated that they do not regard financial benefit to be sufficiently important to justify this practice. Only 19% of the respondents in this sample believed that issuing unwarranted sick certificates to benefit financially was an important reason to issue such certificates.

Both studies show that doctors are unlikely to issue unwarranted sick certificates for financial benefit. The GPs from KwaDukuza suggested that their colleagues do not succumb to pressure from patients to either issue unwarranted medical certificates or to over-service patients for personal financial gain.

The above discussion of the results shows that KwaDukuza GPs perceive their colleagues to be practising ethically, which is in contrast to the findings of the national study.^1^

The majority of GPs reported stress from multiple factors. Almost all doctors reported that external stressors, which include government intervention (100% of respondents indicated that this is a source of considerable stress) and managed care (92%), interfered with the autonomy and professional practice of doctors. Fear of litigation is also a viewed by 72% of the doctors as a significant source of stress. All but one doctor feels that doctors are inadequately remunerated for their work and therefore financial needs also place an additional burden of stress on the GPs. Private GPs need to make a profit to survive in a highly competitive business environment. This together with their reported high stress levels could pressurise doctors into behaving unethically and illegally. GPs from this study perceive their peers to be practising ethically despite the highly stressful environment in which they work.

Both this study on the GPs of KwaDukuza and the Ethics Institute's national study confirm that doctors are facing uncertain times and are under stress from multiple stressors, including financial needs andthe intervention of government or managed care organisations in the practice of medicine.

This small study population of GPs makes the results limited to this population and objectivity and generalisation is therefore limited.

## CONCLUSION

The results differed from that of the national study of the Ethics Institute of South Africa, in which unethical behaviour was highlighted.^1^ KwaDukuza GPs strongly indicated that their colleagues practice ethically. Both studies confirmed that the majority of doctors in South Africa have a greater need for medical ethics education. KwaDukuza GPs perceived their colleagues to be practising ethically in spite of pressure from multiple factors, including demanding patients, government intervention, threat of litigation, managed care intervention in the practice of medicine and constant reduction in their remuneration. The majority of the responding private GPs felt that their peers neither over-service patients nor issue unwarranted medical certificates for personal financial gain. It could be argued that the criticisms of the press are related to the current worldwide problem of the lack of responsible balanced press and media reporting due to financially competitive factors. This study showed findings contrary to the media reports.^8^
